# Long Term Results (>5 Years) in Patients With Peritoneovenous Shunting for Intractable Ascites: Liver Function and Cancer Mortality

**DOI:** 10.1155/1989/48305

**Published:** 1989

**Authors:** Dominique Franco, Jonathan L. Meakins, Andrew Wu, Claude Smadja, Patrick Bonnet, Etienne Gouffier, Bernard Campillo

**Affiliations:** 1 Groupe de Recherche sur la Chirurgie du Foie et de l'Hypertension Portale Hôpital Paul Brousse Villejuif Cédex 94804 France; 2 Groupe de Recherche sur la Chirurgie du Foie et de l'Hypertension Portale Hôpital Louise Michel Evry France; 3 Groupe de Recherche sur la Chirurgie du Foie et de l'Hypertension Portale Hôpital Bicêtre Le Kremlin Bicêtre France; 4 Groupe de Recherche sur la Chirurgie du Foie et de l'Hypertension Portale Hôpital Léon Binet Provins France; 5 Groupe de Recherche sur la Chirurgie du Foie et de l'Hypertension Portale Hôpital Albert Chenevier Créteil France

## Abstract

This report is based on twenty-eight (26%) of 107 patients included in a protocol for prospective evaluation of elective peritoneo-venous shunting for intractable ascites in cirrhosis. These patients had no other procedures and survived more than 5 years after the operation. All patients were free of ascites except one in whom it was mild. One patient refused follow-up. Shunt patency was assessed in 23 patients. In 14 patients (60.9%), the shunt was obstructed and the superior vena cava was occluded in 5 of them. In 9 patients (39.1%), the shunt was still functioning. No clinical or biological parameters differentiated these two groups of patients. Of the 24 patients who were alcoholics, 2 abstained completely and 20 significantly reduced their drinking habits. In 25 patients, the Pugh's score improved and was A at the time of the study. Seven patients (25.9%) developed a malignant tumor of the oro-pharynx or digestive tract, all other patients were alive and in good health. This study suggests that patients with intractable ascites treated by a peritoneo-venous shunt may survive for a long period. In patients abstaining from heavy drinking, it may function as a therapeutic bridge permitting spontaneous improvement of liver function. The risk of supervening neoplasms suggests that a continuous follow-up of these patients is warranted.


*HPB Surgery* 1989, Vol. 1, pp. 185–194
Reprints available directly from the publisher
Photocopying permitted by license only

© 1989 Harwood Academic Publishers GmbH
Printed in Great Britain

### LONG TERM RESULTS (>5 YEARS) IN PATIENTS WITH PERITONEOVENOUS SHUNTING FOR INTRACTABLE ASCITES: LIVER FUNCTION AND CANCER MORTALITY

DOMINIQUE FRANCO[a,b*], JONATHAN L. MEAKINS[b], ANDREW WU[b], CLAUDE SMADJA[c], PATRICK BONNET[c], ETIENNE GOUFFIER[d], and BERNARD CAMPILLO[e]

*Groupe de Recherche sur la Chirurgie du Foie et de l'Hypertension Portale, (a) Hôpital Paul Brousse, Villejuif, (b) Hôpital Louise Michel, Evry, (c) Hôpital Bicêtre, Le Kremlin Bicêtre, (d) Hôpital Léon Binet, Provins, (e) Hôpital Albert Chenevier, Créteil, France*

*(Received 18 July 1988)*

This report is based on twenty-eight (26%) of 107 patients included in a protocol for prospective evaluation of elective peritoneo-venous shunting for intractable ascites in cirrhosis. These patients had no other procedures and survived more than 5 years after the operation. All patients were free of ascites except one in whom it was mild. One patient refused follow-up. Shunt patency was assessed in 23 patients. In 14 patients (60.9%), the shunt was obstructed and the superior vena cava was occluded in 5 of them. In 9 patients (39.1%), the shunt was still functioning. No clinical or biological parameters differentiated these two groups of patients. Of the 24 patients who were alcoholics, 2 abstained completely and 20 significantly reduced their drinking habits. In 25 patients, the Pugh's score improved and was A at the time of the study. Seven patients (25.9%) developed a malignant tumor of the oro-pharynx or digestive tract, all other patients were alive and in good health. This study suggests that patients with intractable ascites treated by a peritoneo-venous shunt may survive for a long period. In patients abstaining from heavy drinking, it may function as a therapeutic bridge permitting spontaneous improvement of liver function. The risk of supervening neoplasms suggests that a continuous follow-up of these patients is warranted.

KEY WORDS: Cirrhosis, Alcoholics, Peritoneovenous shunting, Ascite.

### INTRODUCTION

Intractable ascites is a serious complication of liver cirrhosis. The prognosis is dismal and the one year survival is 4–37.5% [1–4]. There are two available surgical treatments. Portocaval shunt efficiently clears ascites but carries a high postoperative complication rate and a significant incidence of encephalopathy [5–9]. The alternative treatment is peritoneovenous shunting (PVS), which more than ten years after the original description [10] is still not widely accepted [4, 11–14]. The main criticisms of this procedure are: significant operative mortality, high shunt failure rates and poor long term results [4, 12–15]. However, recent improvements in materials [16] and a better understanding of physiological changes after PVS[17–23] have, together with better patient selection, reduced complications [24]. Success rate of PVS has now greatly

*Correspondence and reprints: Dr D. Franco, Hôpital Paul Brousse, 94804 Villejuif Cédex, France improved but long term results are scarce. In 1976, a prospective study of PVS for the treatment of intractable ascites in cirrhotic patients was started [22]. This report examines the general health, outcome of ascites, evolution of liver function and shunt status in patients surviving 5 or more years after PVS.

### PATIENTS AND METHODS

From 1976 to 1981, 107 patients underwent elective peritoneovenous (LeVeen) shunt for the treatment of intractable ascites complicating chronic liver disease. All patients were referred by hepatologists following the failure of intensive medical therapy (hospitalization, salt restriction, intensive diuresis, repeated paracentesis), aggressively pursued for an average of 5.5 months. Details of this study were reported elsewhere [22]. On December 31st. 1986, 33 patients (30.8%) were alive, 7 were lost to follow-up and 67 had died (Table I). Life table analysis is seen in Figure 1. No data are available on PVS patency in these latter 74 patients. Five patients had had in addition a portacaval shunt for recurrent ascites and were therefore excluded from this work. Twenty-eight patients (26.2%) had received no other treatment than PVS for their ascites. One patient refused follow-up. This study comprises the remaining 27 patients, 5 to 10 years after their PVS. There were 17 male and 10 female patients with a mean age of 55 years (range: 37–75 years). Twenty-four patients had alcoholic cirrhosis and 3 had non alcoholic liver disease. At five years, all the patients were clinically assessed for the presence of recurrent ascites. Their alcohol consumption was detailed, by direct questioning not with a standard questionnaire [25]. Full liver function tests were performed and the patients were reclassified according to Child's criteria as modified by Pugh et al [26] and compared to their pre PVS score. Every patient had an ultra-sound. The patency of the shunt (23 patients) and that of the superior vena cava (SVC) (14 patients) were assessed radiologically as previously described [15]. In 4 patients with advanced carcinoma, shuntography was not performed. The clinical course of the 27 patients since 1986 is also reported. The r correlation coefficient was used to study the relations existing between parameters considering each result as a quantitative variable.

### RESULTS

There were no surgical or other complications during the postoperative course of 22 patients (81.5%) after receiving the LeVeen shunt and they remained free of ascites. In 5 patients (18.5%) ascites was controlled only after their shunts had been replaced (7 procedures) because of obstruction or malfunction. Mild ascites, controlled by spironolactone (75 mg/day) persisted in only one patient (3.7%). All other patients (96.3%) were free of ascites both clinically and by ultrasound and none were taking diuretics.

Among the 23 patients who had shuntography, this revealed complete occlusion of the PVS in 14 (60.9%), none of whom had ascites. Superior vena cavogram in 5 showed complete obstruction of the SVC with abundant collateral circulation in all. In 9 patients (39.1%), shuntography demonstrated a patent PVS with flow of ascites. Superior vena cavogram indicated that SVC was patent in all but one. In the latter

**Table I** Causes of death in 67 patients.

| | | *Mean time postop. (months)* |
|---|---|---|
| Postoperative complication | 12 | |
| Hepatocellular carcinoma | 10 | 11 |
| Alcoholic hepatitis | 6 | 23 |
| Hepatic failure | 12 | 18 |
| Infection | 6 | 11 |
| Upper g.i. bleeding | 5 | 6 |
| Miscellaneous causes[a] | 9 | 23 |
| Unknown | 7 | 19 |

[a] Cerebral hemorrhage 2, encephalopathy 2, breast cancer 1, viral hepatitis 1, tuberculosis 1, myocardial infarct 1, bowel obstruction 1.

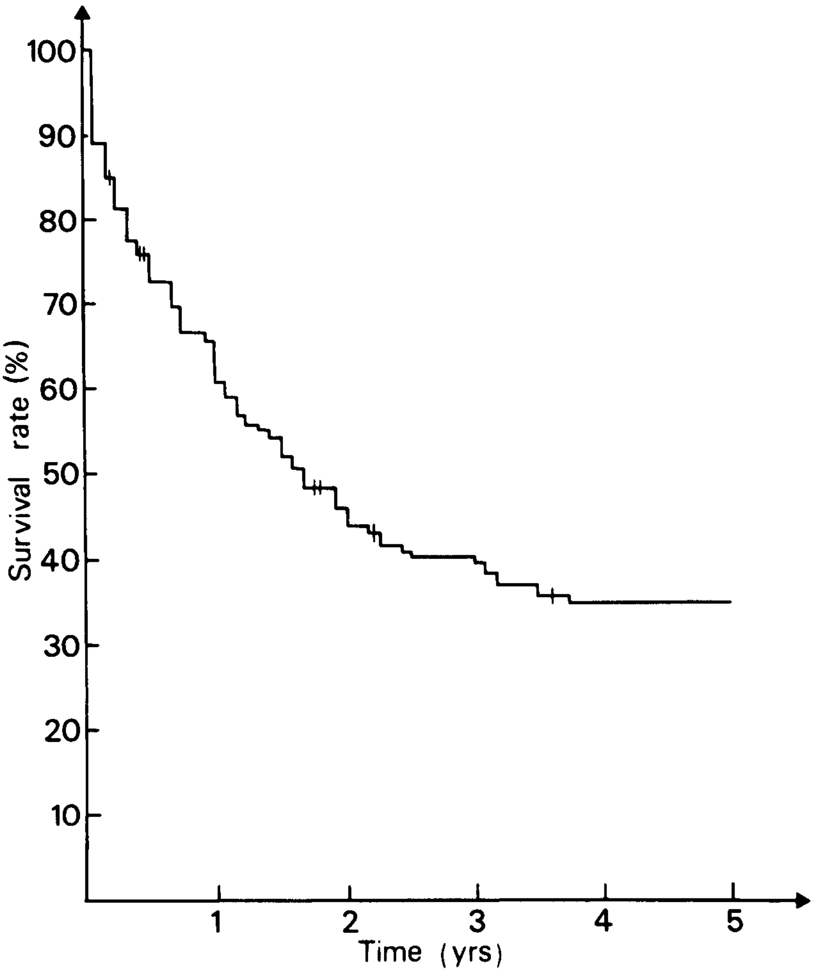

**Figure 1** Life table analysis of all 107 patients who had peritoneovenous shunting for intractable ascites.

patient, SVC had occluded although the tip of the patent venous tubing remained free in the right atrium. Preoperative liver biochemical tests, duration of ascites, and postoperative rate of early shunt occlusion were not different in patients with an occluded and with a patent PVS (Table II). Similarly, there were no identifying characteristics of SVC obstruction.

The changes in liver function are shown in Figure 2. It is apparent that in all the 27 patients, the Pugh score improved after PVS becoming normal in 21; serum albumin improved in 18, prothrombin time in 8 and bilirubin in 2. In one patient mild ascites accounted for a score of 6. In the five others scores of six in 3 and seven in 2 were the result of low serum albumin concentration, slightly elevated serum bilirubin concentration or mild encephalopathy.

**Table II** Relationship between the status of the shunt, duration of intractable ascites, preoperative biochemical liver tests, Pugh's score, and postoperative recurrence of ascites and drinking.

| *Criteria* | *Patients with an occluded shunt (14)* | *Patients with a patent shunt (9)* |
|---|---|---|
| *Preoperative* | | |
| Duration of intractable ascites (months) | 5.2 ± 3.2 | 4.2 ± 3.2 (NS) |
| Serum bilirubin concentration (μM/l) | 18.9 ± 14.6 | 21.6 ± 15.6 (NS) |
| Serum albumin concentration (g/l) | 32.1 ± 3.9 | 32.8 ± 6.1 (NS) |
| ASAT (mU/ml) | 22.2 ± 13.1 | 25.1 ± 14.4 (NS) |
| Prothrombin time (%) | 59.4 ± 17.7 | 59.6 ± 10.4 (NS) |
| Mean Pugh's score | 8.7 ± 1.5 | 8.2 ± 1.4 (NS) |
| *Postoperative* | | |
| Recurrence of ascites requiring replacement of shunt | 1 | 4 (NS) |
| Heavy drinking | 2 | 0 (NS) |

Of the 24 patients who were alcoholics, 2 abstained completely and 20 signficantly reduced their drinking habits. The two who continued to drink were the only patients who remained in Pugh's group B.

Subsequent clinical course, after 1986, is known in all patients. The LeVeen shunt was removed in 5 patients. In 4 patients with an occluded PVS, this was done because of late shunt related complications: two intestinal obstructions, one symptomatic superior vena cava syndrome, and one late neck wound abscess following migration of the venous catheter. After shunt removal, ascites did not reaccumulate in any. In the fifth patient a functional PVS was removed and a portacaval shunt performed before an anterior resection for a low rectal carcinoma [27].

Seven patients (25.9%) developed malignant tumors. Three died, one each from esophageal, laryngeal and tonsillar carcinomas. These were the only deaths in this study group. Three have developed an unresectable hepatocellular carcinoma and the seventh had a rectal carcinoma resected. All other patients were alive without ascites and with exception of one with metastatic hepatoma in apparent good health.

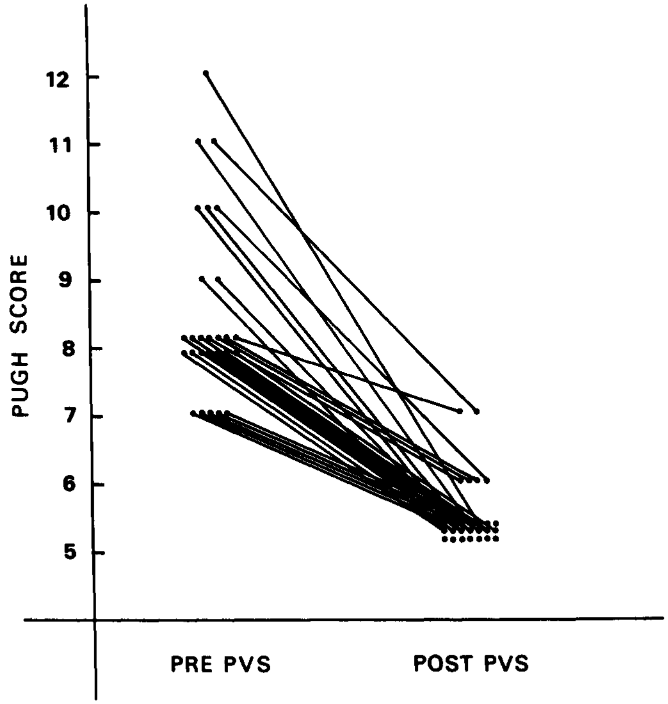

**Figure 2** Changes in Pugh's score 5 years following peritoneovenous shunting in 27 patients with cirrhosis and intractable ascites.

### DISCUSSION

This is the first report on the long-term results of PVS in the treatment of intractable ascites secondary to cirrhosis. All patients alive 5–10 years post PVS except one (96.3%) had their ascites fully controlled by the LeVeen shunt. A few of these patients (18.5%) whose shunt was occluded before the eventual dissolution of ascites had recurrence requiring surgical replacement of the shunt.

At 5 years, 39% of PVS were functioning. No comment can be made about PVS patency in the whole study population. Radiologic demonstration of shunt circulation suggests that ascites was still being formed and its flow was keeping the PVS patent. No ascites was seen on ultrasound indicating immediate clearance. Radioisotope studies were not performed. In these patients, the hemodynamic and metabolic factors contributing to formation of ascites may still persist and the shunt provides a long term supportive measure. This would suggest that persistent ascites formation is not necessarily an indicator of poor prognosis in patients with cirrhosis.

On the other hand, 61% of patients had occluded shunts. The absence of ascites demonstrates that its formation had ceased. In these patients their initial intractable ascites was eventually correctable and PVS provided a therapeutic bridge until it stopped forming. We have found no biological or clinical indicator, either prior to or following shunting to distinguish between these 2 groups of patients.

It is noteworthy that PVS not only relieves ascites but that it may allow the impaired liver to recover spontaneously. Indeed, after shunting, twenty-five of the 27 patients improved their Pugh's score to class A. Several hypotheses may be advanced to explain this recovery of liver function. The removal of ascites and its accompanying symptoms allows re-establishment of an adequate dietary intake. This improvement in nutritional status favours a return to normal or at least improvement to an optimal liver function [(28)]. A reduction in the abdominal pressure following the shunting of ascites may favourably alter the distribution of blood flow within the liver. Also hepatic and systemic hemodynamic changes resulting from reinfusion of ascites may improve perfusion and encourage regeneration of the liver parenchyma. This has yet to be confirmed [(29)]. Most patients abstained from alcohol after PVS. The cessation of insults to the liver by alcohol abuse may also result in an improvement in liver function [(30)]. The 2 patients who remained in Pugh's class B following PVS continued to have massive alcohol intake. This is in accordance with previous reports suggesting that decompensation of cirrhosis is enhanced by heavy drinking [(31)]. While spontaneous regression of tense ascites has been observed on a long-term basis in almost 100% of patients with alcoholic abstinence, it occurred in only 17% of patients with persistent drinking [(2)]. PVS may increase the rate of patients with favourable evolution and shorten the delay of recovery.

The deaths occurring after 5 years of follow-up are related to extra-hepatic cancers. This is contrary to many reports of intermediate term mortality where liver failure has been the principle mode of death [(4, 22, 32)]. The observation in these long-term survivors of a remarkably increased incidence of cancer of the oro-pharynx (tobacco and alcohol related) or digestive tract (25.9%) has not been reported. These figures are higher than those reported by Ginès et al [(33)] who studied the natural history of compensated cirrhosis. These data suggest that patients with cirrhosis, who have had intractable ascites successfully controlled, require long-term follow-up as they appear susceptible to a variety of neoplasms.

### *References*

1. Stone, W.D., Islam, N.R.K. and Paton, A. The natural history of cirrhosis. *Quat J Med 1968;* **145:** 119–32.
2. Capone, R.R., Buhac, I., Kohberger, R.C. and Balint, J.A. Resistant ascites in alcoholic liver cirrhosis. Course and prognosis. *Dig Dis Sci 1978;* **23**: 867–71.
3. Wapnick, S., Grosberg, S.J. and Evans, M.I. Randomized prospective matched pair study comparing peritoneovenous shunt and conventional therapy in massive ascites. *Br J Surg 1979;* **66**: 667–70.
4. Bories, P., Compean-Garcia, D. Michel, H. et al. The treatment of refractory ascites by the LeVeen shunt. A multi-center controlled trial (57 patients). *J Hepatol 1986;* **3:** 212–18.
5. Barker, H.G. and Reemtsma, K. The portacaval shunt operation in patients with cirrhosis and ascites. *Surgery 1960;* **48**: 142–54.
6. Welch, H.F., Welch, C.S. and Carter, J.H. Prognosis after surgical treatment of ascites: results of side-to-side shunt in 40 patients. *Surgery 1964;* **56**: 75–82.
7. Burchell, A.R., Rousselot, L.M. and Panke, W.F. A seven-year experience with side-to-side portacaval shunt for cirrhotic ascites. *Ann Surg 1968;* **168**: 655–70.
8. Orloff, M.J. Pathogenesis and surgical treatment of intractable ascites associated with alcoholic cirrhosis. *Ann NY Acad Sci 1970;* **170**: 213–38.
9. Franco, D., Vons, C., Traynor, O. Smadja, C. Should portal systemic shunt be re-considered in the treatment of intractable ascites in cirrhosis? *Arch Surg 1988*, **123**; 987–992.
10. Leveen, H.H., Christoudias, G. and Moon, I.P., et al. Peritoneovenous shunting for ascites. *Ann Surg 1974;* **180**: 580–99.
11. Greig, P.D., Langer, B. Blendis, L.M., et al. Complications after peritoneovenous shunting for ascites. *Am J Surg 1980;* **139**: 125–31.
12. Foley, W.J., Elliott, J.P. and Smith, R.F., et al. Central venous thrombosis and embolism associated with peritoneovenous shunts. *Arch Surg 1984;* **119**: 713–20.

13. Gleysteen, J.J. and Klamer, T.W. Peritoneovenous shunts: predictive factors of early treatment failure. *Am J Gastroenterol 1984;* **79**: 654–58.
14. Smith, R.E., Nostrant, T.T. Eckhauser, F.E. et al. Patient selection and survival after peritoneovenous shunting for non malignant ascites. *Am J Gastroenterol 1984;* **79**: 659–62.
15. Smadja, C., Tridart, D. and Franco, D. Recurrent ascites due to central venous thrombosis after peritoneojugular (LeVeen) shunt. *Surgery 1986;* **100**: 535–40.
16. Franco, D., Labianca, M. Smadja, C. et al. A titanium catheter tip for peritoneovenous shunts. *Artif Organs 1988;* **12**: 81–82.
17. Ansley, J.D., Bethel, R.A., Bowen, P.A. and Warren, W.D. Effect of peritoneovenous shunting with the LeVeen valve on ascites, renal function and coagulation in six patients with intractable ascites. *Surgery 1978;* **83**: 181–87.
18. Darsee, J.R., Fulenwider, J.T. Rikkers, L.F. et al. Hemodynamics of LeVeen shunt pulmonary edema. *Ann Surg 1981;* **194**: 189–92.
19. Tawes, R.C., Sydorak, G.R. Kennedy, P.A. et al. Coagulopathy associated with peritone-ovenous shunting. *Am J Surg 1981;* **142**: 51–55.
20. Ragni, M.V., Lewis, J.H. and Spero, J.A. Ascites-induced LeVeen shunts coagulopathy. *Ann Surg 1983;* **198**: 91–95.
21. Franco, D., Smadja, C. and Descorps Declere, A. Coagulation defects following peritoneovenous shunt. In: Fondu P and Thys O: hemostatic failure in liver disease, Raven Press, Bruxelles 1984; 108–20.
22. Smadja, C. and Franco, D. The LeVeen shunt in the elective treatment of intractable ascites in cirrhosis . A prospective study on 140 patients. *Ann Surg 1985;* **201**: 488–93.
23. Biagini, J.R., Belghiti, J. and Fekete, F. Prevention of coagulopathy after placement of peritoneovenous shunt with replacement of ascitic fluid by normal saline solution. *Surg Gynecol Obstet 1986;* **163**: 315–18.
24. Hillaire, S., Labianca, M. Smadja, C. et al. Dérivation péritonéoveineuse dans la cirrhose: résultats d'une étude prospective sur les facteurs d'amélioration du pronostic. Gastroentérol Clin Biol 1988; **12** 681–6.
25. Bernadt, M.W., Taylor, C. Mumford, J. et al. Comparison of questionnaire and laboratory tests in the detection of excessive drinking and alcoholism. *Lancet 1982;* **1**: 325–28.
26. Pugh, R.N.H., Murray-Lyon, I.M. Dawson, J.L. et al. Transection of the esophagus for bleeding esophageal varices. *Br J Surg 1973;* **60**: 646–49.
27. Lerolland, B., Kahwaji, F. Smadja, C. et al. Management of colorectal cancer in patients with cirrhosis and a LeVeen shunt. *Int Surg 1987;* **72**: 93–95.
28. Blendis, L.M., Harrison, J.E. Russel, D.M. et al. Effects of peritoneovenous shunting on body composition. *Gastroenterology 1986;* **90**: 127–34.
29. Vons, C., Hadengue, A. Lee, S.S. et al. Hémodynamique des malades atteints de cirrhose avec une ascite. Différences entre les répondeurs et les non répondeurs au traitment médical. Effect du shunt péritonéo-veineux. *Gastroentérol Clin Biol 1987;* **11** (supp): 191.
30. Pares, A., Caballeria, J. Bruguera, M. et al. Histological course of alcoholic hepatitis. Influence of abstinence, sex and extent of hepatic damage. *J Hepatol 1986;* **2**: 33–42.
31. Borowsky, S.A., Strome, S. and Lott, E. Continued heavy drinking and survival in alcoholic cirrhotics. *Gastroenterology 1981;* **80**: 1405–9.
32. Fulenwider, J.T., Smith, I.I.I. R.B. Redd, S.C. et al. Peritoneovenous shunts. Lessons learned from an eight-year experience with 70 patients. *Arch Surg 1984;* **119**: 1133–37.
33. Gines, P., Quintero, E. Arroyo, V. et al. Compensated cirrhosis: natural history and prognostic factors. *Hepatology 1987;* **7**: 122–28.

### COMMENTARY

This interesting report from Franco and his colleagues reports the investigation and clinical status of 28 patients who have survived for more than 5 years after the placement of a peritoneo-venous shunt (PVS). The majority of these patients appear to have had a good quality of life and had shown an improvement in liver function (Pugh Score) after the PVS had been established. This improvement may have a number of causative agents because 22 of the 24 alcoholics in this group had more or less reformed their drinking habits.

It is important to evaluate this paper in the light of the authors' previous report of 140 patients (86% alcoholic liver disease) undergoing PVS after an average of 4 months intensive medical management. 14 patients died in the perioperative period (10%). The mortality rate could be related to the level of liver dysfunction (25% mortality for severe liver dysfunction). Of the 126 survivors 38 (30.5%) developed recurrent ascites by 2 years and in nearly all this was due to venous obstruction or obstruction to the valve. There was no recurrence of ascites after 2 years.

16 patients suffered gastrointestinal haemorrhage mainly from varices and this was associated with a mortality risk of 31%. 8 of these patients underwent portocaval shunts (12 emergency, 6 elective). 11 patients developed peritoneal infection and 7 of these patients died.

69 patients died during the period of observation which extended from 2 to 45 months.

In this current paper there are 28 five year survivors from 107 population. It is not possible to completely marry the patient population but it would appear that the overall 5 year survival is of the order of 25%. It is interesting that of the longer term 57 survivors in the original report, 11 required to undergo portocaval shunts either for variceal bleeding or recurrent ascites and 5 lived more than 5 years. Survival times were recorded at 1 year of 77% for patients with good liver function, 61.3% for those with moderate liver function. At 3 years, the survival figures appear to be 47% for patients with good liver function and only 20% for those with poor liver parameters. It is likely, therefore, that the bulk of the 5 year survivors are those who had good or improved liver function.

The doubtful role of the PVS in maintaining the patients long term is emphasised by the fact that 14 of 23 patients who were investigated had evidence of shunt occlusion, and 5 had evidence of superior vena obstruction.

It is interesting to speculate on the results of randomised study between portocaval shunt (PCS) and PVS for intractable ascites. Although it may be too optimistic, an operative mortality of 10% for elective PCS could be achieved in specialist units and long term is likely to be associated with a reduced risk of infection and variceal haemorrhage. The authors appear to have achieved this quality of performance for PCS in their own patients with intractable ascites. It is unlikely that the longterm survival will be worse than 25% reported here and could be better if the alcoholic patients alter their drinking patterns. These gains have to be counterbalanced by an increased encephalopathy rate. It is probable that in patients with severe liver dysfunction no form of treatment other than hepatic replacement will be effective but in the remainder there is no clear advantage to the PVS over conventional portosystemic shunting.

This report has thrown up an interesting observation that of the 5 year survivors, 7 patients (25.9%) developed a malignant tumour of the digestive tract. In their original report an additional 12 patients died within 45 months of hepatocellular carcinoma and one of breast cancer. It is interesting to speculate on aetiological factors including viral and clinical carcinogens. However in population which includes so many alcoholics, it would be hard to dissociate smoking related disease from the question. The authors are right to draw our attention to this added lethal risk in patients who have apparently stabilised their chronic liver disease.

Geoffrey R. Giles M.D.
Professor of Surgery
The University of LEEDS
Clinical Sciences Building
St James's University Hospital
LEEDS LS9 7TF
GREAT BRITAIN

### INVITED COMMENTARY

In this retrospective review, the authors analyze 28 patients who survived at least 5 years from a group of 107 patients undergoing LeVeen shunt from 1976 to 1981. All but one were free of ascites and there was a significant improvement in the Pugh Score. Twenty-three patients underwent shuntography and 39% had a patent shunt. SVC thrombosis was present in six patients (26%). Subsequent to 1986, 7 of these patients have developed malignant tumours.

This is one of the first reports of the long-term survival following peritoneovenous shunting. To date, most reports have concentrated on the early mortality in which one year actuarial survival rates have ranged from 25–80% [1,2,3] and depend on the patient population being shunted [1,2,4,5]. In this report, the 26% actual five-year survival is very low and represents a decrease from the overall 41% survival of 140 patients previously reported by this same group (1). This likely reflects the natural history of the underlying liver disease.

This study confirms the overall improvement in patients who do well with peritoneovenous shunting. The improvement in Pugh score and control of ascites in all but one patient are consistent with previous reports of improved nutritional status and well-being with a successful shunt [4,6,7].

Of interest is the high incidence of shunt occlusion (60% of patients studied), yet these patients were free of ascites. Combined with the 26% survival, it calculates that only 12% of the 107 shunts that were placed were patent at the five-year point. The high incidence of SVC thrombosis (26%) is also significant and represents a serious complication for the patient who might be considered a candidate for liver transplantation in the future.

The identification of an occluded shunt in the absence of ascites has significant implications regarding the pathogenesis of ascites. The LeVeen shunt has been used as a tool to unravel the etiological factors in the ascites associated with cirrhosis and portal hypertension [8]. In general, these have been studies of the acute effects of peritoneovenous shunting although longer term follow-up studies have recognized a persistent inability of the cirrhotic patient to handle a sodium load. In this five-year follow-up study, 60% of survivors had an occluded shunt, yet were free of ascites without diuretic therapy. The implication is that the sodium-retaining lesion that these patients had preoperatively is reversible in the long term. Whether this primary lesion is peripheral arterial vasodilation and vascular unresponsiveness remains to be determined [9]. The authors could identify no difference in the preoperative status of patients with an occluded shunt and patients with a patent shunt; it is open to speculation whether those patients with a patent shunt had the same reversible lesion, and whether the shunt was "functionally patent".

The high rate of late cancers is interesting. However, the numbers are so small that it is difficult to regard peritoneovenous shunting as being a significant cancer risk, since the incidence of esophageal, laryngeal and tonsillar carcinomas are more frequent in the alcoholic population than in the general population, and hepatocellular carcinoma is more common in cirrhotics.

This long-term study has confirmed that when patients do well following peritoneovenous shunting, the results are very good. The 26% actual five-year survival and the 40% patency rate in survivors combined with the well-recognized 10–25% operative mortality rate, all emphasize the high risk nature of the patient population.

Paul D. Greig, M.D.
Toronto General Hospital
Toronto, Ontario M56 2CH
Canada

### *References*

1. Smadja, C. and Franco, D. The LeVeen shunt in the elective treatment of intractable ascites in cirrhosis. A prospective study of 140 patients. *Annals of Surgery, 1985;* **201** (4): 488–93
2. Bories, P., Garcia-Compean, D., Michel, H., Bourel, M., Capron, J.P., Gauthier, A., Lafon, J., Levy, V.G., Pascal, J.P., Quinton, A. et al. The treatment of refractory ascites by the LeVeen shunt. A multi-centre controlled trial (57 patients). *Journal of Hepatology, 1986;* **3**(2): 212–8
3. Greig, P.D., Langer, B., Blendis, L.M. and Taylor B.R. Complications after peritoneovenous shunting for ascites. *American Journal of Surgery, 1980;* **139**: 125–131
4. Fulenwider, J.T., Smith, R.B., Redd, S.C., Ansley, J.D., Henderson, J.M., Millikan, W.F., Galambos, J.T. and Warren, W.D. Peritoneovenous shunts. Lessons learned from an eight-year experience with 70 patients. *Archives of Surgery, 1984;* **119**(10): 1133–7
5. Gleysteen, J.J. and Klamer, T.W. Peritoneovenous shunts: predictive factors of early treatment failure. *American Journal of Gastroenterology, 1984;* **79**(8): 654–8
6. Blendis, L.M., Harrison, J.E., Russell, D.M., Miller, C., Taylor, B.R., Greig, P.D. and Langer B. Effects of peritoneovenous shunting on body composition. *Gastroenterology, 1986;* **90**(1): 127–34
7. Franco, D., Charra, M., Jeambrun, P., Belghiti, J., Cortesse, A., Sossler, C. and Bismuth, H. Nutrition and immunity after peritoneovenous drainage of intractable ascites in cirrhotic patients. *American Journal of Surgery, 1983;* **146**(5): 652–7
8. Blendis, L.M. The use of peritoneovenous shunting in unravelling the pathogenesis of ascites in cirrhosis. *Israel Journal of Medical Sciences, 1986;* **22**(2): 78–80
9. Schrier, R.W., Arroyo, V., Bernardi, M., Epstein, M., Henriksen, J.H. and Rodes, J. Peripheral arterial vasodilation hypothesis; a proposal for the intitiation of renal sodium and water in cirrhosis. Hepatology, 1988; **8**: 1151–57

*Accepted by S. Bengmark on 11 November 1988.*

